# Interspecific variation in leaf traits, photosynthetic light response, and whole-plant productivity in amaranths (*Amaranthus* spp. L.)

**DOI:** 10.1371/journal.pone.0270674

**Published:** 2022-06-30

**Authors:** Mildred Osei-Kwarteng, Emmanuel Ayipio, Dany Moualeu-Ngangue, Gerhard Buck-Sorlin, Hartmut Stützel

**Affiliations:** 1 Institute of Horticultural Production Systems, Leibniz University Hannover, Hannover, Germany; 2 Department of Horticulture, Faculty of Agriculture, Food and Consumer Sciences, University for Development Studies, Nyankpala, Tamale, Ghana; 3 CSIR-Savannah Agricultural Research Institute, Nyankpala, Ghana; 4 Auburn University, Department of Horticulture, Auburn, Alabama, United States of America; 5 IRHS, INRAE, Institut Agro, Université d’Angers, Beaucouzé, France; Bangabandhu Sheikh Mujibur Rahman Agricultural University, BANGLADESH

## Abstract

Photosynthetic light response curve parameters help us understand the interspecific variation in photosynthetic traits, leaf acclimation status, carbon uptake, and plant productivity in specific environments. These parameters are also influenced by leaf traits which rely on species and growth environment. In accessions of four amaranth species (*Amaranthus*. *hybridus*, *A*. *dubius*, *A*. *hypochondriacus*, and *A*. *cruentus*), we determined variations in the net photosynthetic light response curves and leaf traits, and analysed the relationships between maximum gross photosynthetic rate, leaf traits, and whole-plant productivity. Non-rectangular hyperbolae were used for the net photosynthesis light response curves. Maximum gross photosynthetic rate (*P*_gmax_) was the only variant parameter among the species, ranging from 22.29 to 34.21 μmol m^–2^ s^–1^. Interspecific variation existed for all the leaf traits except leaf mass per area and leaf inclination angle. Stomatal conductance, nitrogen, chlorophyll, and carotenoid contents, as well as leaf area correlated with *P*_gmax_. Stomatal conductance and leaf nitrogen explained much of the variation in *P*_gmax_ at the leaf level. At the plant level, the slope between absolute growth rate and leaf area showed a strong linear relationship with *P*_gmax_. Overall, *A*. *hybridus* and *A*. *cruentus* exhibited higher *P*_gmax_ at the leaf level and light use efficiency at the whole-plant level than *A*. *dubius*, and *A*. *hypochondriacus*. Thus, *A*. *hybridus* and *A*. *cruentus* tended to be more efficient with respect to carbon assimilation. These findings highlight the correlation between leaf photosynthetic characteristics, other leaf traits, and whole plant productivity in amaranths. Future studies may explore more species and accessions of *Amaranthus* at different locations or light environments.

## Introduction

The response of photosynthesis to light is vital in predicting carbon fixation in the field because leaf photosynthesis rate is mainly influenced by the variations in light under field conditions [1, 2]. Photosynthetic light response curves describe the relationship between leaf net photosynthesis rate and the photosynthetic photon flux density (PPFD) incident on the leaf surface [1, 3, 4]. They reveal the current acclimation state of a leaf, which helps to understand carbon uptake and productivity of plants in specific environments [3, 5, 6]. Leaf photosynthesis response to light can be described by several models such as the rectangular and non-rectangular hyperbola or exponential functions and their modifications [1, 3–9] The non-rectangular hyperbola model is among the most commonly used due to its broad applicability to C_3_ and C_4_ species [5, 7, 10–12]. The parameters of these models are often included in canopy photosynthesis and ecosystem models of plant productivity and gas exchange [1, 12, 13].

Some leaf traits are indicators of plant acclimation to the growth light environment [14, 15]. Interspecific and intraspecific photosynthetic variation among and across species can be explained by leaf traits such as nitrogen content, chlorophyll (Chl) content, leaf dry mass per unit area (LMA), leaf angle, and stomatal conductance (*g*_s_) [1, 3, 12, 16–19].

Plants develop photosynthetic characteristics and other leaf traits depending on the local light environment [20–23]. Most of our knowledge on photosynthetic light response curves and leaf traits stems from plants grown under controlled conditions, which is insufficient to assess plant acclimation to natural growth light conditions [22, 24, 25]. Plants grown under natural light conditions experience rapid fluctuations in light due to solar movement, weather, and canopy characteristics [21, 25–28], and variations in light can occur at timescales ranging from seconds to weeks [24, 29, 30]. Consequently, acclimation to natural light conditions may result in different physiological, biochemical, and morphological properties of plants [22, 24]. Acclimation of plants to alterations in natural light environments depends on the plant species (phenotypic plasticity) and the environment to which it is adapted [26, 28, 30–35]. Acclimation may occur at the leaf and whole-plant levels [21, 22, 25, 30, 36]. Plants grown under natural light conditions may combine the characteristics of both low light and high light-grown plants, allowing them to use light efficiently [24, 37]. For instance, some classical responses of plants to high light conditions include: high chlorophyll (Chl) *a* to Chl *b* ratio (Chl *a*/*b*) [22–24, 28, 33, 38–43]; low total Chl content [38, 42–45]; high LMA [22, 24, 38]; low Chl *b* content; higher leaf photosynthesis rates [24, 33, 38, 43, 46, 47], erect leaf orientation [22], and the opposite is experienced under low light conditions [22, 46, 48]. Quantum yield of CO_2_ uptake is at its upper limit under low light conditions and also often shows no significant difference between high and low light-grown plants [4, 23, 32]. The convexity factor tends to be higher in low light and vice versa, with intermediate values found under medium light conditions [11, 23].

Amaranths (*Amaranthus* spp. L.) are NAD-dependent malic enzyme (NAD-ME) subpathway C_4_ type, annuals, herbaceous, dicotyledonous, or rarely short-lived perennials with worldwide distribution. The genus consists of about 87 species originating from the tropics [49, 50]. Consequently, amaranths perform best in warm climates and thrive under high irradiance levels [50–54]. They grow well at day temperatures above 25°C and night temperatures not lower than 15°C. The genus includes vegetable (*A*. *tricolor* L., *A*. *blitum* L., *A*. *dubius* L, *A*. *cruentus* L., and *A*. *viridis* L.), grain (*A*. *hypochondriacus* L., *A*. *cruentus* L., and *A*. *caudatus* L.), weed (*A*. *palmeri*, *A*. *retroflexus*, and *A*. *hybridus*) and ornamental species (brightly coloured *A*. *tricolor*, *A*. *caudatus* and *A*. *hypochondriacus*) [53, 55–57]. The leaves of all the species can be consumed depending on regional preferences [53, 58]. Variations in photosynthetic capacity among 12 amaranth species were found to be positively correlated with stomatal conductance, nitrogen and Chl contents, and LMA [16]. However, no comparative studies have been conducted in amaranth species under natural growth light conditions on the photosynthesis light response, leaf traits, and how interspecific variations in photosynthetic light response curve parameters at the leaf level are related to other leaf traits and whole-plant productivity.

The objectives of this study were: 1) to determine the variations in the net photosynthetic light response (*P*_N_/*I*) curves and leaf traits, 2) to explain the variations in the variant parameter [i.e., maximum gross photosynthetic rate (*P*_gmax_)] by the leaf traits, and 3) to explore how the variation in *P*_gmax_ correlates with growth rate and leaf area at the whole-plant level in four amaranth species. We hypothesised that there is variation in the leaf photosynthetic light response curves and leaf traits among the amaranth species. The differences in the leaf photosynthetic light response curves correlate with leaf traits and whole plant productivity.

Parameters of the non-rectangular hyperbola such as the maximum gross photosynthetic rate (*P*_gmax_), apparent quantum yield at zero PPFD [α (*I*_0_)], convexity (θ) and dark respiration rate (*R*_D_) were estimated for each gas exchange measurement. Key leaf traits such as stomatal conductance (*g*_s_), nitrogen per unit leaf area (N_a_), leaf dry mass per unit area (LMA), Chlorophyll (Chl, Chl *a*, Chl *b*), carotenoid (Car) content, leaf area (LA) and leaf inclination angle were measured.

Under the natural growth light environments of this study, interspecific variation in *P*_gmax_ and some key leaf traits were observed among the amaranth species. Interspecific variation in *P*_gmax_ was mainly explained by *g*_s_ and N_a_, while at the whole-plant level, *P*_gmax_ was strongly influenced by the variations in light use efficiency (slope of the natural logarithm of absolute growth rate and leaf area per plant).

## Materials and methods

### Plant materials

Accessions of four cultivated Amaranthus species, namely, *A*. *hybridus* (‘IP7’; weed), *A*. *dubius* (‘Mombo 2’; vegetable), *A*. *hypochondriacus* (‘TZ-SMN-102’; grain), and *A*. *cruentus* ‘Ex-Zim/Madiira 1’; vegetable) were obtained from the Asian Vegetables Research and Development Centre (AVRDC), Arusha, Tanzania (S1 Fig). All the species were reported to have been collected (i.e., country of collection) from Africa, but the origin of *A*. *hybridus* is unknown; *A*. *dubius* and *A*. *hypochondriacus* are from Tanzania, and *A*. *cruentus* from Zimbabwe. The number of days to flowering (from sowing to 50% inflorescence when characterised in Tanzania) are 31, 25, 35, 73 for *A*. *hybridus*, *A*. *dubius*, *A*. *hypochondriacus*, and *A*. *cruentus*, respectively [59]. The four amaranth species were chosen because of their genetic diversity (variation), contrasting plant architecture (morphology), and since they are taxonomically well characterised and commercially important in East Africa [52, 53, 57, 60–63].

### Experimental site, cultivation, and experimental design

The experiment was conducted at the Institute of Horticultural Production Systems, Leibniz University of Hannover, Germany (52.2°N, 9.7°E). Seeds were sown on March 18, 2014, in trays with Potgrond (peat) tray substrate (Klasmann-Deilmann, Geest, Germany) and raised in a growth cabinet at 22°C/ 20°C, day and night temperature, respectively. The nutrient composition of the Potgrond substrate was: 210 mg L^–1^ N, 240 mg L^–1^ P_2_O_5_, 270 mg L^–1^ K_2_O 100 mg L^–1^ Mg and 150 mg L^–1^ S, with a pH of 6.0. Vigorous plants were transplanted into 10 litre pots (diameter of ca. 26 cm (top) & 19 cm (bottom); height, 24 cm) of a 1:1 mixture of sand and Potgrond peat-based substrate. Pots were arranged at a spacing of 60 cm and 40 cm, between and within rows, respectively. Plants were grown under natural light conditions in the glasshouse without supplementary light from lamps. However, the temperature was regulated as 24/22°C, day/night air temperatures, respectively. Ventilators were opened when the air temperature was higher than 26°C. Plants were watered daily with 0.5–1% (50-100g/100L H_2_O) Ferty^®^ 2 MEGA [16+6+26 (+3.4)] to avoid nutrient and water stress. The experiment was conducted as a randomised complete block design and replicated four times. Photosynthesis measurements were conducted on two of these replications.

The weather data were recorded at 12-minutes intervals by the Institute’s weather station, situated at 36 m from the glasshouse.

### Gas exchange and leaf trait measurements

Data on the leaf traits and photosynthesis gas exchange were collected on May 7, 12, and 20, 2014, corresponding to 50, 55, and 63 days after sowing (DAS), respectively (S1 Table). All gas exchange measurements were made on the uppermost youngest fully expanded leaves. These leaves were selected from different plants at each measurement date. A portable photosynthesis gas exchange system (LI-6400, LI-COR, Inc., Lincoln, NE, USA) equipped with a red/blue light-emitting diode (LED) light source was used for the simultaneous measurement of photosynthesis and stomatal conductance. Measurements were made at 400 μmol mol^-1^ ambient atmospheric CO_2_ concentration, a flow rate of 300 μmol s^-1^, mean leaf temperature of 25°C ± 1.6°C, and a vapour pressure deficit (VPD) of 1.3 ± 0.3 kPa. Measurements were made from 09:00 h to 15:00 h, at photosynthetic photon flux density (PPFD) levels of 0, 50, 100, 150, 200, 250, 300, 400, 450, 500, 600, 800, 1,000 1,200 and 1,500 μmol (photon) m^–2^ s^–1^. The light curves were started at the lowest PPFD. Leaves were adapted for at least 5–20 min per light level to ensure that photosynthesis and stomatal conductance were stable before data logging.

Following the gas exchange measurements, the same youngest fully expanded leaves were used for leaf trait measurements. Leaves were placed on ice in a cool box and taken to the laboratory to determine leaf area and leaf pigments. Leaf area was measured with a leaf area meter LI-3100 (LI-COR, Lincoln, NE, USA). Leaves were oven-dried at 70°C for at least 96 h and weighed to determine their dry mass.

Chl and Car contents were determined in a whole-pigment extract of leaf tissues by UV-VIS spectroscopy [40]. The absorbance of the extract was measured at 470.0 nm, 648.6 nm, and 664.2 nm for the calculation of the Chl *a*, Chl *b* and Car contents [40]. Nitrogen content was determined by the Nelson and Sommers [64] procedure.

Leaf inclination angle was obtained with a three-dimensional (3D) digitiser (Fastrak, Polhemus Inc., Colchester, VT, USA) [65, 66]. Leaf inclination angle is expressed in the range from zero to 180 degrees, where zero indicates an upward vertical leaf and 180 a downward dropping leaf [66].

### Estimation of growth rate and the relationship between growth rate and leaf area

The oven-dried weight of the above-ground plant parts (shoots) at five measurement dates (28, 42, 50, 55, and 63 DAS) (S1 Table) and the intervals (14, 8, 5, and 8 days) between the measurement dates were used to calculate the growth rate. Absolute growth rate (AGR; increment in dry weight per unit time) of the plants across the measurement intervals was calculated as

$$AGR=\frac{{W_{2}-W}_{1}}{t_{2}-t_{1}}$$ (1)

where W_1_ and W_2_ are the dry weights at the beginning and the end of the interval at times t_1_ and t_2_, respectively [67]. Plant growth rate and total leaf area per plant were assessed to explore the variation in *P*_gmax_ at the whole-plant level. In crops, light interception is often exponentially related to leaf area [68–70], and growth can be considered the product of light interception and light use efficiency [69, 70]. Thus, we can expect a linear relationship between the natural logarithm of absolute growth rate and leaf area per plant, where the slope of the relationship should indicate light use efficiency.

### Photosynthesis model

The four-parameter non-rectangular hyperbola leaf photosynthesis model [4] was employed in this study. We used a Microsoft Excel routine (Solver) to estimate the four key parameters of the *P*_N_/*I* curve. The routine uses the non-linear least square curve fitting procedure (generalised reduced gradient method) [4].

The model is of the form:

$$P_{\text{N}}=\frac{\alpha (I_{0})\;\text{x}\;I\;+\;P_{\text{gmax}}-\;\sqrt{\alpha (I_{0})\;\text{x}\;I\;+{\left(P_{\text{gmax}}\right)}^{2}-\;4\theta \;\text{x}\;\alpha (I_{0})\;\text{x}\;I\;\text{x}\;P_{\text{gmax}}}}{2\theta }-R_{\text{D}}$$ (2)

Where *I* [μmol photon) m^–2^ s^–1^] is the photosynthetic photon flux density (PPFD); *P*_gmax_ [μmol (CO_2_) m^–2^ s^–1^] is the asymptotic estimate of the maximum gross photosynthetic rate; α (*I*_0_) [μmol (CO_2_) μmol^-1^ (photon)], is the apparent quantum yield at *I* = 0 (based on incident light) [4, 37, 71]; θ [dimensionless] is the convexity or rate of bending of the curve (the ratio of physical-to-total resistance (carboxylation resistance + physical resistance [8, 72]) and *R*_D_ [μmol (CO_2_) m^–2^ s^–1^] is dark respiration (measured at *I* = 0, intercept on the Y-axis). *P*_N_ [μmol (CO_2_) m^–2^ s^–1^] is the net photosynthetic rate. It is important to note that the definition of apparent quantum yield, α (*I*_0_), does not correspond to the original concept of the maximum quantum yield (α) of photosynthesis light response. The maximum quantum yield, α, is usually defined as the slope of the curve at the linear portion in the range of PPFD between 0 and 200 μmol (photon) m^–2^ s^–1^ [4, 73–76]. In contrast, α (*I*_0_) is the derivative of the four-parameter non-rectangular hyperbola at *I* = 0 [4]. Thus α (*I*_0_) is instead the maximum value of quantum yield higher than any point on the *P*_N_*/I* curve [4]. The parameters of the non-rectangular hyperbola were estimated separately for each leaf.

### Statistical analysis

A two-way ANOVA was conducted on the parameters of the *P*_N_/*I* curve and the leaf traits to test the effects of the species and measurement dates. Significant differences between means were determined using the Tukey honest significant difference (THSD) test at a 5% probability level. It is also important to report some effect size measures that indicate whether the observed statistical differences among groups are of practical significance. For a two-way ANOVA and small sample size, the effect size measure omega squared (ω^2^) is recommended [77–82]. ω^2^ also determines the percentage of the variation in the dependent variable attributable to the individual independent factors (i.e., species and measurement dates) [78]. ANOVA and ω^2^ were both computed using JMP Pro software version 13 (SAS Institute Inc., 2016). Correlation (Pearson’s) analysis was used to establish the association between leaf traits and maximum gross photosynthetic rate (*P*_gmax_). Linear regression analysis was also used to establish the relationship between the leaf traits and *P*_gmax_.

## Results

### Environmental variables

**Fig 1:**
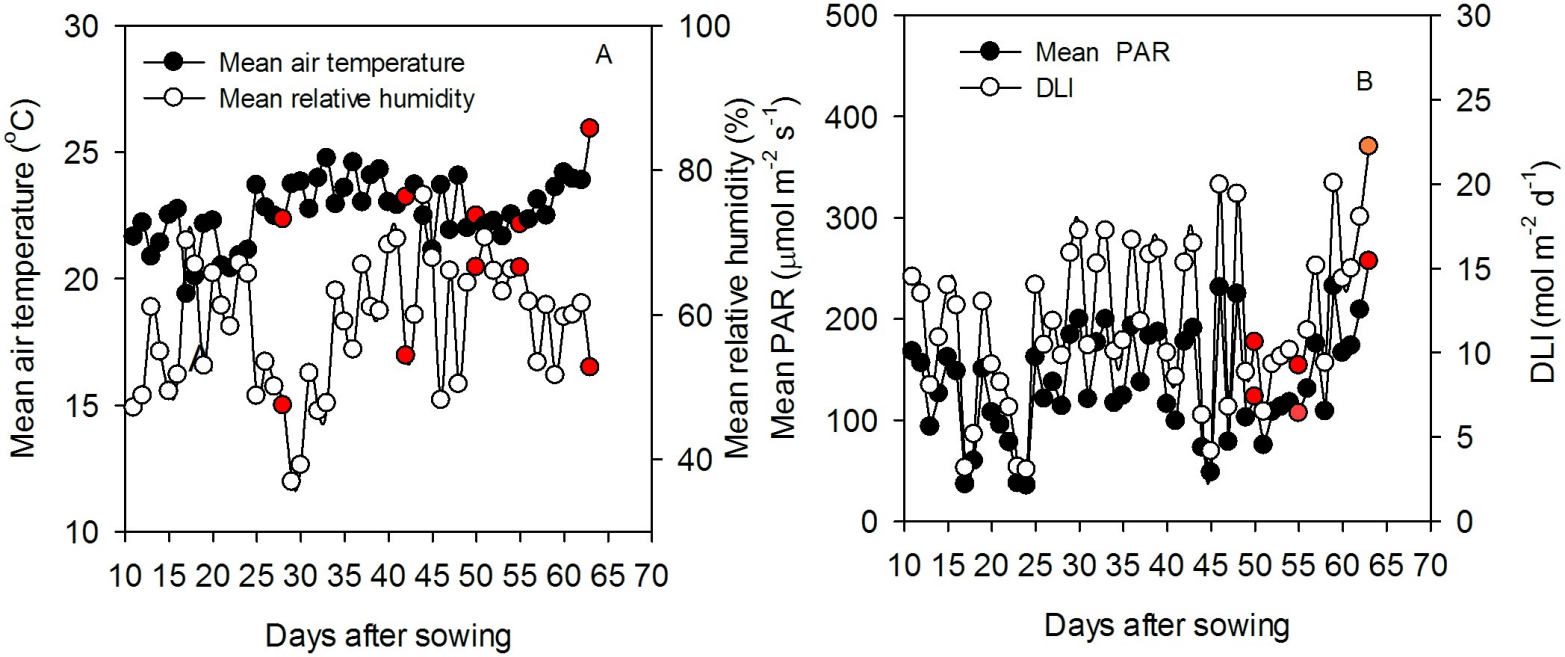
Weather variables during the growing period. **(A)** Mean air temperature and relative humidity. (B) Daily mean photosynthetically active radiation (PAR) and daily light (PAR) integral (DLI). The red spots in A correspond to the five days (28, 42, 50, 55, and 63 DAS) for the growth rate measurements and the red spots in B correspond to the three days (50, 55, and 63 DAS) for the photosynthetic light response and leaf trait measurements. DAS denotes days after sowing.

The mean air temperature, mean relative humidity, mean daily photosynthetically active radiation (PAR) and mean daily light integral (DLI) during the growing period are shown in Fig 1 below.

The range of the daily mean PAR and DLI in the glasshouse during the growth period were 35.2 to 257 μmol m^–2^ s^–1^ and 3.1 to 22.2 mol m^–2^ d^–1^, respectively. The mean temperature and relative humidity during the whole growth period were 22.7°C and 58.4%, respectively. Light intensities at and in the five- or ten-day intervals before measurements were highest for the third measurement date (May 20, 2014; 63 DAS), followed by the first (May 7, 2014; 50 DAS) and the second (May 12, 2014; 55 DAS) (Table 1).

**Table 1:** Daily light integral (DLI) at three measurement dates, means across five (5 d) and ten days (10 d) prior, and the average from transplanting to the three measurement dates (TM).

Measurement	Date (2014)	Days after sowing (DAS)	Daily Light Integral (DLI) (mol PAR m^-2^ d^-1^)			
-	-	-	Daily	Mean (5 d)	Mean (10 d)	Mean (TM)
1	07 May	50	10.61	11.61	11.48	11.37
2	12 May	55	9.23	9.25	10.42	11.11
3	20 May	63	22.20	16.50	14.05	11.80

### Net photosynthetic-light response (*P*_N_/*I*) curves

**Fig 2:**
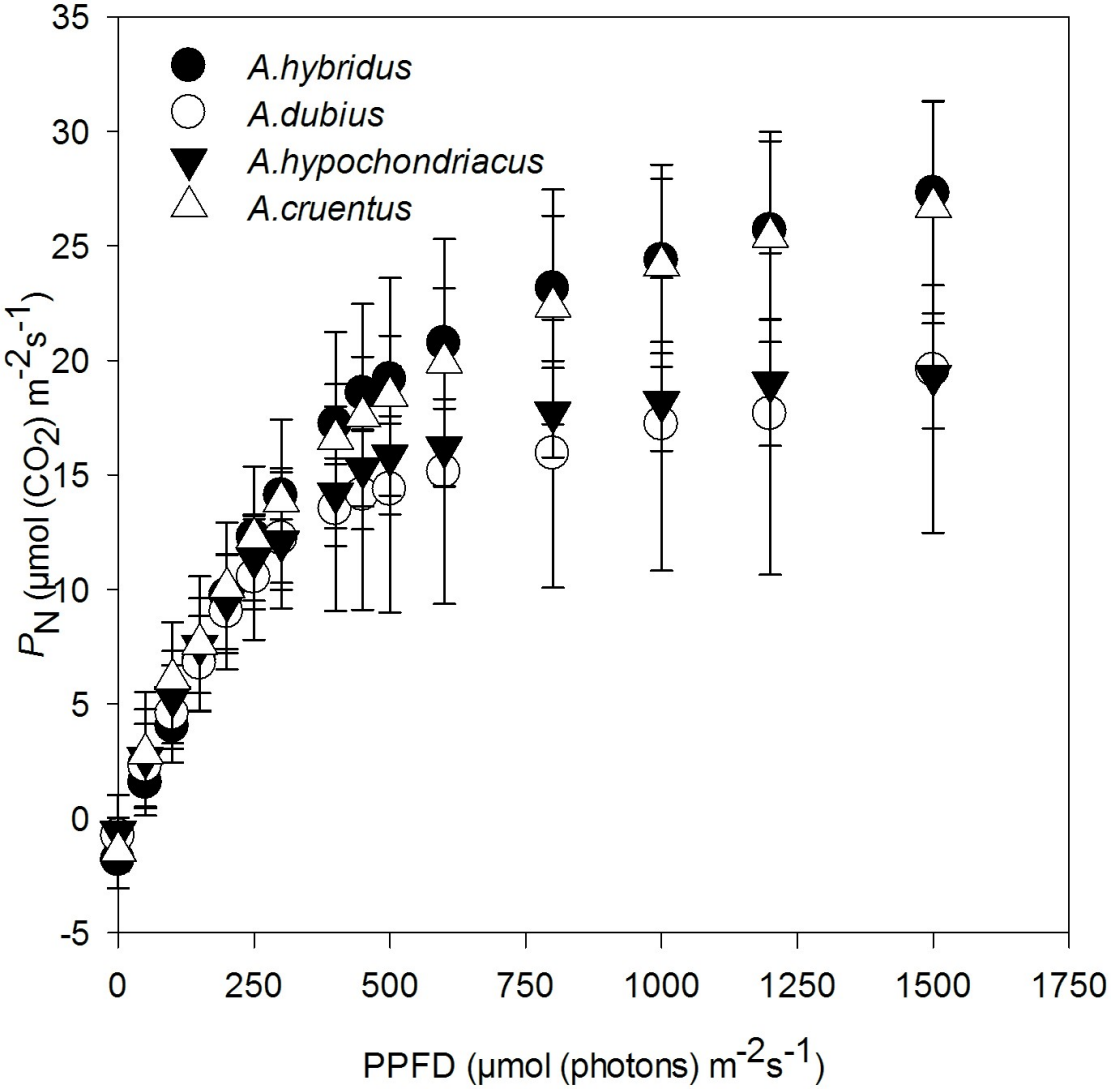
The response of net photosynthetic rate, *P*_N_, to photosynthetic photon flux density (PPFD) on new fully expanded leaves of four amaranth species. The symbols for each species represent the mean pooled data of six light response curves (*n* = 6, two from each of the three measurement dates) since there was no measurement date effect on the light response curves. Lines joining the points were omitted for clarity. Bars represent ± SD. Measurement conditions were leaf temperature of 25°C, CO_2_ concentration of 400 μmol mol^-1^, average relative humidity of 60–70%, and a vapour pressure deficit of 1.3 ± 0.3 kPa.

Non-rectangular hyperbolae described the net photosynthesis light response (*P*_N_/*I*) of the four amaranth species well at each measurement date (S2 Fig). The two-way ANOVA test showed no main effect of measurement date and an interactive effect of measurement dates and species on all the model parameters (S2 Table). The maximum gross photosynthesis rate (*P*_gmax_) was the only parameter that differed (p<0.001) among the species. Due to the lack of measurement date effects, the data on the *P*_N_/*I* curves were pooled for the species (Fig 2).

The mean separation test on *P*_gmax_ categorised the species into two; high (*A*. *cruentus* and *A*. *hybridus)* and low *P*_gmax_ (*A*. *dubius and A*. *hypochondriacus*). Apparent quantum yield at PPFD of zero, dark respiration, and convexity were not significantly different among the species (Table 2).

**Table 2:** Means and confidence intervals (95%) for apparent quantum yield at zero PPFD (α(*I*_0_)), convexity (θ), dark respiration rate (*R*_D_), and the mean comparison (Tukey honest significant test) of species effect on maximum gross photosynthesis (*P*_gmax_).

Species	*P*_*gmax*_ (μmol (CO_2_) m^–2^ s^–1^)	α(*I*_0_) (μmol (CO_2_) μmol (photon) –1)	*R*_D_ (μmol (CO_2_) m^–2^ s^–1^)	θ
*A*.*hybridus*	32.57^A^ (± 4.16)	0.07 (± 0.02)	2.05 (± 1.00)	0.66 (± 0.36)
*A*.*dubius*	22.29^B^ (± 4.16)	0.08 (± 0.03)	1.09 (± 1.00)	0.41 (± 0.36)
*A*.*hypochondriacus*	21.86^B^ (± 4.16)	0.08 (± 0.02)	1.02 (± 1.00)	0.49 (± 0.36)
*A*.*cruentus*	34.21^A^ (± 4.16)	0.07 (± 0.02)	1.12 (± 1.00)	0.34 (± 0.36)

Mean values of *P*_gmax_ with different superscripts are significantly different at the 5% level. *n* = 24

### Interspecific variations in leaf traits

The two-way ANOVA showed a significant species effect on most of the leaf traits except for LMA and leaf inclination angle (S3 Table). There was a measurement date effect on both total Chl and Chl *b* (S4 Table). Significant interactions between measurement date and species were also found for Chl *a* and Chl *b* ratio (Chl a/b) (S5 Table). *A*. *hybridus* had the highest values for all pigments, while *A*. *cruentus* had the highest N_a_ and *g*_s_ values (Table 3).

**Table 3:** Mean comparison (Tukey Honest Significant Difference) and 95% confidence intervals of amaranth species for stomatal conductance (*g*_s_), nitrogen content per unit area (N_a_), chlorophyll (Chl), and carotenoids (Car) contents. *n* = 24 (*g*_s_), *n* = 48 for the rest of the traits.

Species	*g*_s_ (mol m^–2^ s^–1^)	N_a_ (g m^–2^)	Chl *a* (mmol m^–2^)	Chl *b* (mmol m^–2^)	Total Chl (mmol m^–2^)	Car (mmol m^–2^)
*A*. *hybridus*	0.20^A^ (± 0.07)	1.95^AB^ (± 0.34)	0.55^A^ (± 0.11)	0.15^A^ (± 0.03)	0.71^A^ (± 0.13)	0.23^A^ (± 0.04)
*A*.*dubius*	0.14^AB^ (± 0.07)	1.85^AB^ (± 0.34)	0.38^B^ (± 0.11)	0.09^C^ (± 0.03)	0.46^B^ (± 0.13)	0.16^B^ (± 0.04)
*A*.*hypo-chondriacus*	0.10^B^ (± 0.07)	1.66^B^ (± 0.34)	0.43^B^ (± 0.11)	0.10^BC^ (± 0.03)	0.53^B^ (± 0.13)	0.18^B^ (± 0.04)
*A*. *cruentus*	0.19^A^ (± 0.07)	2.03^A^ (± 0.34)	0.46^AB^ (± 0.11)	0.11^B^ (± 0.03)	0.58^AB^ (± 0.13)	0.19^B^ (± 0.04)

The stomatal conductance (*g*_s_) presented here was measured at the maximum light level (1500 μmol m^–2^ s^–1^).

### Relation between maximum gross photosynthesis rate (*P*_gmax_) and leaf traits

Positive correlations were found between *P*_gmax_ and leaf pigments except for Chl *b*. Correlations were highly significant for *P*_gmax_ and stomatal conductance and nitrogen per unit area (S6 Table).

**Fig 3:**
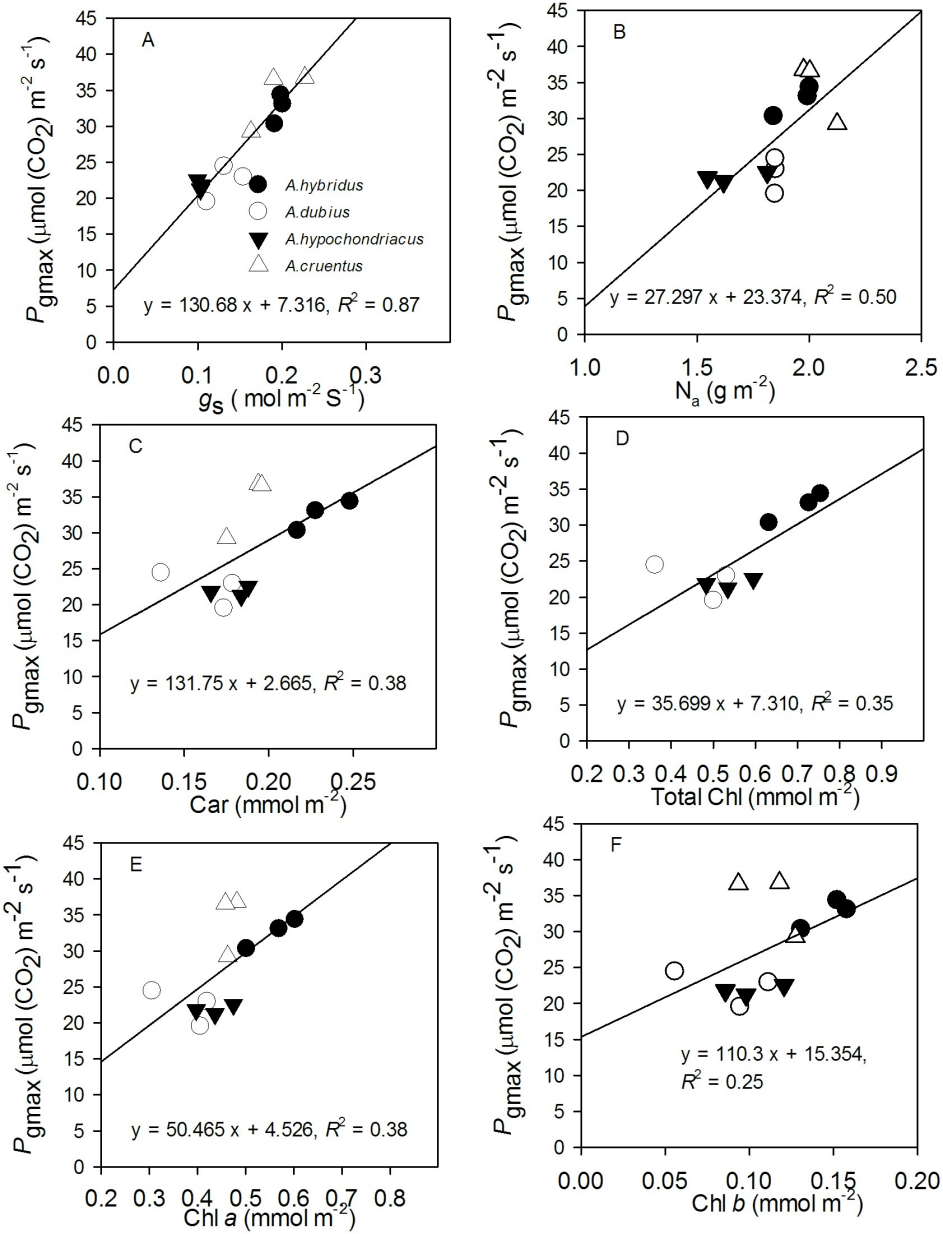
Relationship between leaf traits and maximum gross photosynthetic rate (*P*_gmax_) for four amaranth species (*A*. *hybridus*, *A*. *dubius*, *A*. *hypochondriacus*, and *A*. *cruentus*). *A*–*F* (stomatal conductance (*g*_s_) measured at the maximum PPFD of 1500 μmol (photons) m^–2^ s^–1^, nitrogen per unit area (N_a_), carotenoids (Car), Chlorophyll (Chl *a*, total Chl, Chl *b*). The three data points for each species represent the means of the variables at the three measurement dates (50, 55, and 63 days after sowing). Measurement conditions for the gas exchange measurements were leaf temperature of 25°C, CO_2_ concentration of 400 μmol mol^-1^, average relative humidity of 60–70%, and a vapour pressure deficit of 1.3 ± 0.3 kPa.

Accordingly, *P*_gmax_ showed a strong positive linear relationship with *g*_s_ measured at 1500 μmol (photons) m^–2^ s^–1^. A positive linear relationship was also found between N_a_, leaf pigments, and *P*_gmax_ (Fig 3).

**Fig 4:**
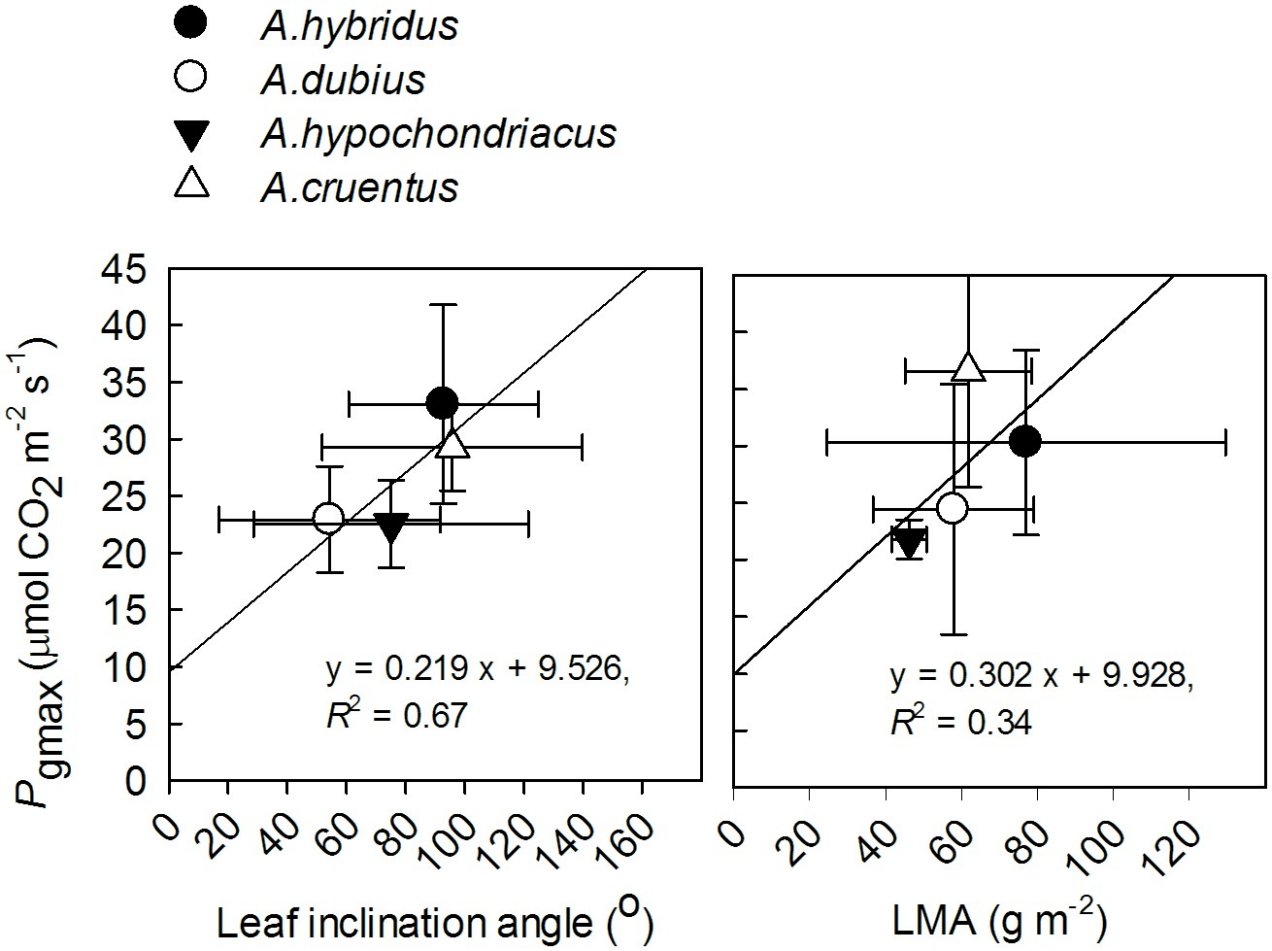
Relation between maximum gross photosynthesis rate (*P*_gmax_) and leaf inclination angle (left) and leaf dry mass per unit area (LMA) (right) at measurement date 1 (50 DAS) and 3 (63 DAS), respectively. The four data points represent the means of each species. Bars represent the ± SD of the means. DAS denotes days after sowing.

Leaf inclination angle showed a positive linear relationship with *P*_gmax_ only at the first measurement date (50 DAS). LMA exhibited a weak linear relationship with *P*_gmax_ only at the third measurement date (63 DAS; Fig 4).

### Relationship between whole-plant variables and maximum gross photosynthesis rate (*P*_gmax_)

**Fig 5:**
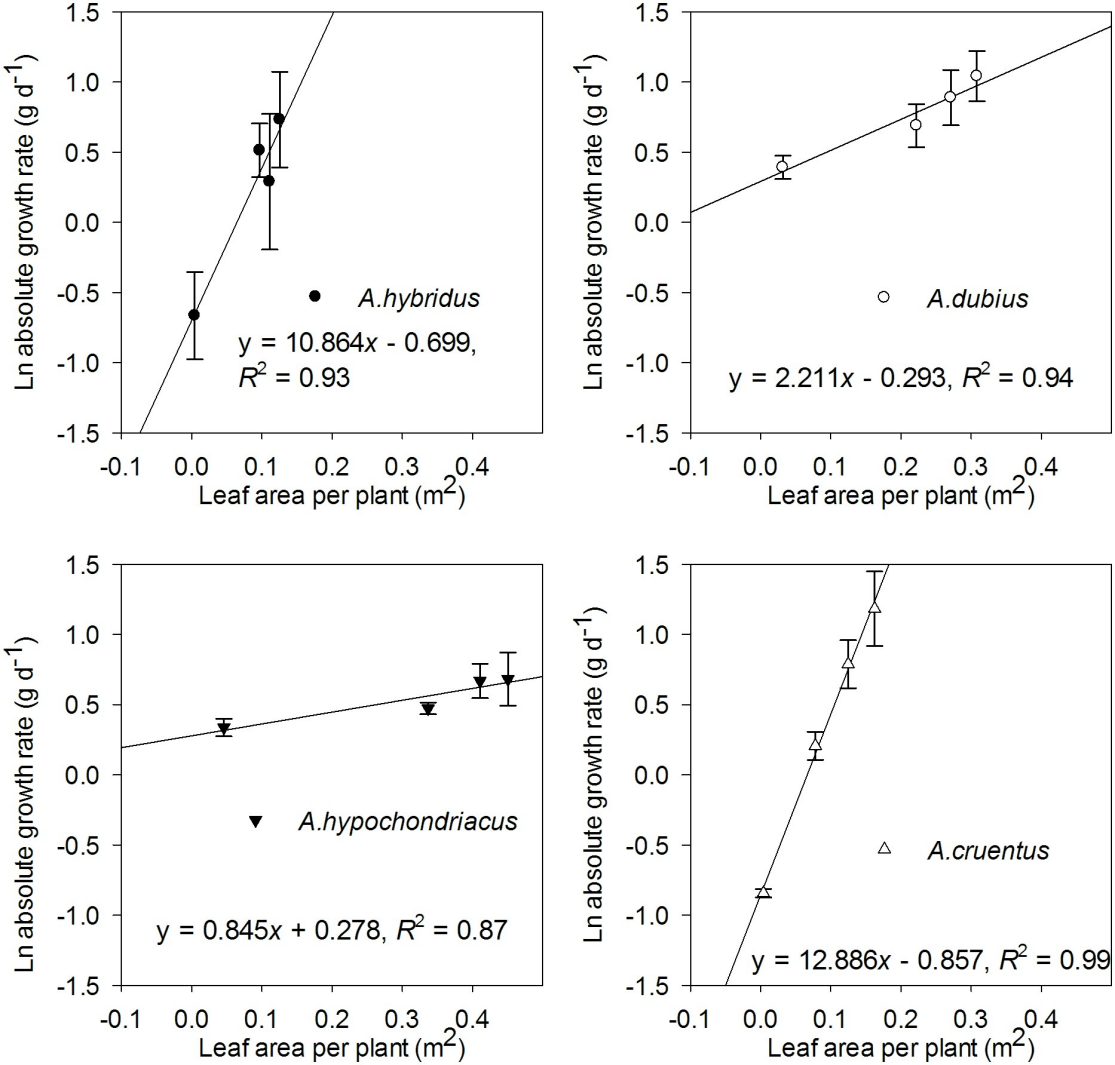
The relationship between the natural logarithm of absolute growth rate (AGR) and leaf area per plant of four amaranth species (*A*. *hybridus*, *A*. *dubius*, *A*. *hypochondriacus*, and *A*. *cruentus*). The four data points for each species represent the means of the variables at the four measurement date intervals. Bars represent the ± SD of the means.

A strong linear relationship was found between the natural logarithm of absolute growth rate and leaf area per plant for all the species (Fig 5).

The slopes of the linear relationship varied among the species (Table 4). The differences in slopes corroborate the pattern of the variation in the *P*_gmax_. Accordingly, *A*. *cruentus* and *A*. *hybridus* had high and similar slopes while *A*. *dubius* and *A*. *hypochondriacus* exhibited low slopes (Table 4).

**Table 4:** The slope of the linear relationship between the natural logarithm of absolute growth rate (AGR) and leaf area per plant.

Species	Slope (g d^-1^m^-2^)
*A*.*hybridus*	11.32^A^
*A*.*dubius*	2.26^B^
*A*.*hypochondriacus*	0.84^B^
*A*.*cruentus*	12.48^A^

**Fig 6:**
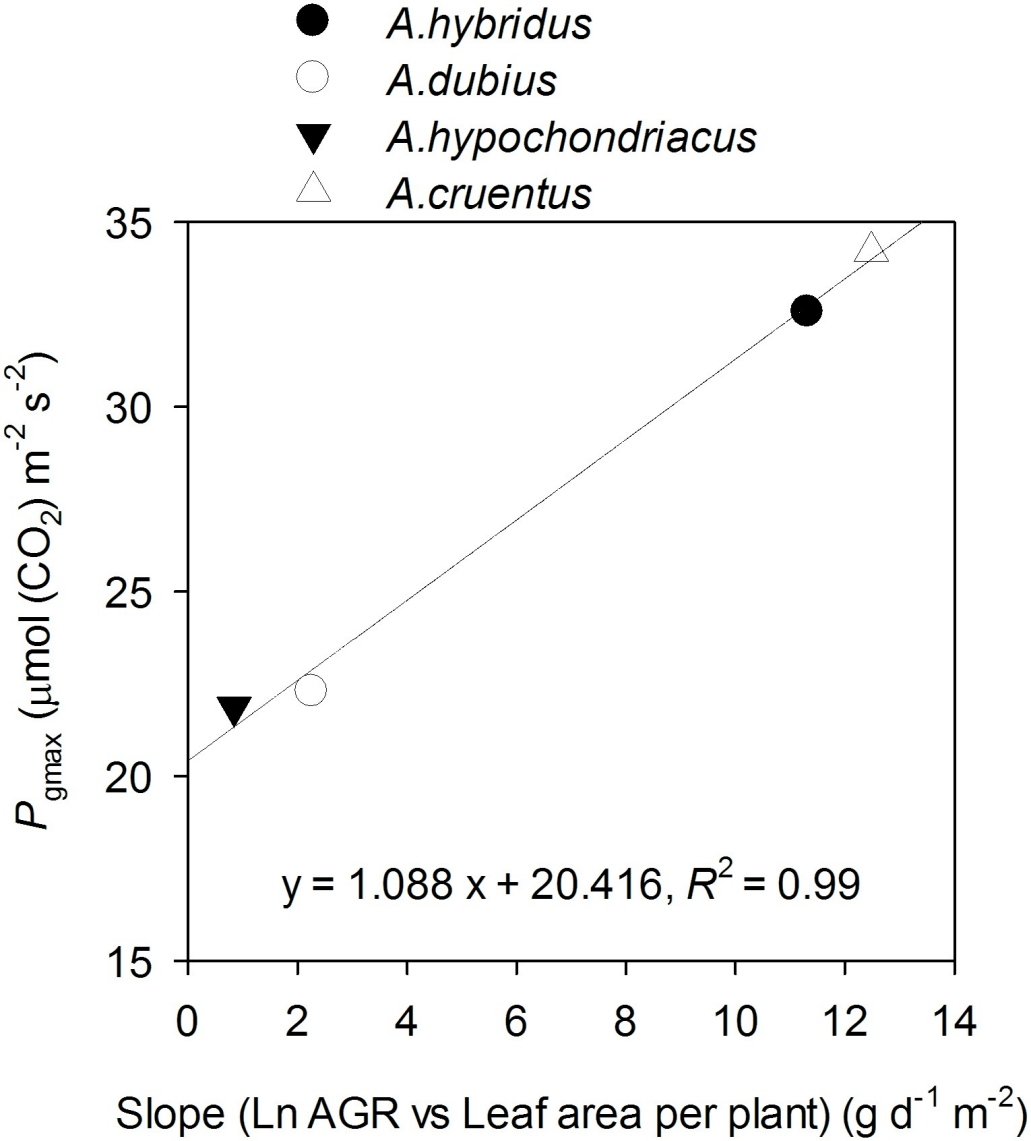
The relationship between maximum gross photosynthetic rate (*P*_gmax_) and the slope between the natural logarithm of absolute growth rate and leaf area per plant.

There was a strong linear relationship between the slope and *P*_gmax_ (Fig 6).

## Discussion

In the present study, we investigated the net photosynthetic light response (*P*_N_/*I*) curves, leaf traits, and productivity at the whole-plant level in four amaranth species. We observed variation in maximum gross photosynthetic rate, *P*_gmax_, and some key leaf traits among the species. At the leaf level, stomatal conductance predominantly explained this variation. At the whole-plant level, the slope of the linear relationship between the natural logarithm of absolute growth rate and leaf area per plant varied among the species and was strongly correlated with *P*_gmax_.

*P*_gmax_ is widely used for the ecophysiological characterisation of plant species and comparative analysis of growth conditions [83]. Many plant anatomical or morphological, chemical (e.g. Chl content per leaf area), physiological (e.g. photosynthesis rate per leaf area) and growth traits (e.g. growth rate) are better related to the daily light integral (DLI; i.e., the PAR integrated over the day) than to instantaneous or peak values of PAR at any specific moment in time [35, 84]. Hence the average DLI during an experimental treatment can be used to quantify the light intensity experienced by plants [35]. The average DLIs calculated from the onset of the experiment in the glasshouse to each measurement date were similar for the three measurement dates (Table 1). The similarity in the average DLI presumably is why measurement dates had no significant effect on the parameters of the *P*_N_/*I* curves [35, 84]. Hence, the differences in *P*_gmax_ observed among the species represented the species’ innate acclimated photosynthetic performance under the conditions of growth [75, 85, 86]. The observed *P*_gmax_ are in the range reported in previous studies for amaranths [45, 75, 87]. Also, the trend of the variations in *P*_gmax_ among the species agrees with the findings of [16]. These researchers found that weedy amaranths such as *A*. *hybridus* and fast-growing amaranths such as *A*. *cruentus* exhibit a higher photosynthesis rate than grain (*A*. *hypochondriacus*) and vegetable amaranths (*A*. *dubius*).

Quantum yields of normal healthy leaves do not differ among species under non-stressed growth conditions [36, 44, 88, 89]. Ehleringer et al. [90] also found that the quantum yield of both C_4_ and C_3_ species is not dependent on the growth light and temperature conditions. Our values are similar to the theoretical quantum yield for C_4_ plants (i.e., 0.07, when there is no CO_2_ leakage from the bundle sheath to the mesophyll and 0.063 μmol (CO_2_) μmol (photon) –^1^ when there is leakage) [90–92]. Our values are also consistent with those of NAD-ME enzyme type C_4_ grass species, *Sporobolus cyrptandrus*, *Panicum virgatum*, and maize (Zea mays) [0.06 μmol (CO_2_) μmol (photon) –^1^] [7, 90]. Harley and Ehleringer [75] determined the quantum yields of four amaranth species, including three species used in this study. They also found no significant difference among the species.

The coefficient θ represents the photosynthetic efficiency in the intermediate light range above the linear section determined by the maximum quantum yield. Photosynthesis in the intermediate light range is most efficient when θ is high [11]. Commonly observed leaf θ values range from 0.5 to 0.99 [11, 13, 71, 93], and two of our values were in this range. Nevertheless, all our values (Table 2) were in the range observed in C_4_ plants [7, 93].

*R*_D_ is known to vary depending on the acclimation state or ambient light environment [94, 95]. The observed *R*_D_ was reasonably proportional to the observed *P*_gmax_, indicating the coupling relationship between photosynthesis rate and respiration rate [96].

### Interspecific variation in leaf traits

The species showed interspecific variation in leaf traits except for LMA and leaf inclination angle. The variation in *g*_s_, N_a_, and Chl content agrees with the findings of [16], who found interspecific variation in 12 amaranth species. LMA indicates the position of species along a gradient of resource-rich to resource-poor environments [97]. Average DLI during plant growth determines the LMA of plants [84, 98]. In our study, the average DLI received by the plants at the measurement dates were similar (Table 1), which is consistent with the similar LMA among the species and measurement dates. At the species level, *A*. *hybridus* and *A*. *cruentus* had higher total Chl content, Chl *a*, N_a_, and *g*_s_ than *A*. *dubius* and *A*. *hypochondriacus*, which corroborated their higher *P*_gmax_ (Tables 2 and 3) [2, 16, 99, 100].

Plants grown under natural light conditions possess high acclimation capacity to alterations in light, which is measurable in the pigment composition of thylakoids [24, 41]. Thus under natural growth light conditions, plants combine the characteristics of low and high light-grown plants for an efficient utilisation of light [24]. The observed decrease in total Chl and Chl *b*; and increase in Chl *a*/*b* ratio at the last measurement date corroborated the known properties of plants grown in natural fluctuating light conditions [24, 41]. Our data shows that the PAR and DLI prior to, or on, the first and second measurement dates (7 and 12 May 2014; 50 and 55 DAS) were similar and lower than at the last measurement date (20 May 2014; 63 DAS) (Table 1 and Fig 1B). Both Chl *b* and total Chl are known to decrease in high growth light environments due to the reduced proportion in light-harvesting complex proteins in favour of electron transport, photophosphorylation, and carbon fixation components [33, 38, 41, 43–45, 48]. Chl *a*/*b* ratio is a primary index of the acclimation to light, which measures the proportion of light-harvesting complex to other Chl components [101]. A higher ratio occurs in high growth light environments where Chl *a* content or the photosystem I chlorophyll increases and the proportion of light-harvesting chlorophyll *a*/*b*-protein complex decreases [41, 42, 48]. The increase in the Chl *a*/*b* ratio was species-specific, as noted by [28, 42]. *A*. *dubius* and *A*. *cruentus* showed a significant increase in Chl *a*/*b* ratio at the last measurement date. In contrast, *A*. *hybridus* and *A*. *hypochondriacus* maintained a similar Chl *a*/*b* ratio across the measurement dates (S5 Table). This suggests that *A*. *dubius* and *A*. *cruentus* could reduce their light-harvesting complex proteins when the growth light environment improved [29, 41–43]. The observed range of values was similar to reported values for C_4_ plants, including amaranths [41, 42]. The central role of Chl *b* and Car is to broaden the absorption spectrum of plants for maximal light capture [33, 35, 48]. Among the species, *A*. *hybridus* differed in Chl *b* and Car content suggesting a broader spectrum for maximal light capture (Table 3).

The interspecific variation observed in *g*_s_, in the present study, ranging from 0.14 to 0.20 mol m^–2^ s^–1^, is similar to values in [34] found in 12 amaranth species (0.17 to 0.26 mol m^–2^ s^–1^). Their values were slightly higher than ours, probably due to the differences in the leaf temperatures (30°C and 25°C) used for the gas exchange measurements. Liu and Stützel [51] reported variation in *g*_s_ between 0.35 mol m^–2^ s^–1^ and ca. 0.60 mol m^–2^ s^–1^ among four genotypes of amaranth. Our values are comparatively low, apparently due to the low temperatures (24/22°C, day/night) during our study compared to the high temperatures (30/20°C, day/night) in their research. Urban et al. [102] showed that *g*_s_ increased by about 40% when the temperature was increased by 10°C at a constant VPD of 1 kPa in both broadleaf and coniferous species. Low light environments can also contribute to stomatal closure [103, 104].

### Exploring the relationship between leaf traits and the maximum gross photosynthesis rate (*P*_gmax_)

Interspecific variation in *P*_gmax_ was directly related to biochemical and physiological leaf traits such as stomatal conductance, nitrogen, and Chl content. In contrast, structural leaf traits such as leaf thickness were not directly involved [16]. Our observation confirms these findings. The interspecific variation in *P*_gmax_ was also mainly explained by stomatal conductance and nitrogen content, although leaf pigments were also associated (Fig 3). Many C_3_ and C_4_ plant species showed similar positive linear relationships [16, 50, 99, 105–107]. According to von Caemmerer et al. [103], the striking correlation between photosynthetic capacity and *g*_s_ maintains the Ci (intercellular CO_2_ concentration) /Ca (ambient CO_2_ concentration) ratio constant when photosynthetic capacity is modulated in the long-term by growth conditions. The fairly strong positive relationship between *P*_gmax_ and leaf inclination angle at the first measurement date (Fig 4) suggests that as leaf inclination angle increased (i.e., became more horizontal from 50° to 100°), *P*_gmax_ also increased. Also, leaf angles tend to be more horizontal under low light environments to increase the efficiency of direct light absorption [22, 108]. Plants maximise their total net photosynthetic gain by maximizing whole plant PPFD absorption and photosynthetic light use efficiency via simultaneous adjustments in leaf angle and leaf photosynthetic capacity [109].

### Plant leaf area, growth rate, light use efficiency, and *P*_gmax_

Plant productivity, especially in low light environments, depends on the net photosynthetic rate of individual leaves but is also strongly dependent on the total leaf area displayed for light interception [36]. Our findings demonstrate that the slope between the natural logarithm (ln) of absolute growth rate and leaf area per plant, representing light use efficiency (LUE), was strongly associated with the variation in *P*_gmax_. The two species (*A*. *cruentus* and *A*. *hybridus*) with a higher *P*_gmax_ showed higher slopes (Fig 5). Thus, *A*. *cruentus* and *A*. *hybridus* were more efficient in converting light energy into photosynthates [110].

## Conclusion

Our data revealed interspecific variation in the maximum gross photosynthetic rate (*P*_gmax_), stomatal conductance, nitrogen content, and leaf pigments per unit area among four amaranth species. The variation in *P*_gmax_ was mainly explained by stomatal conductance and nitrogen content at the leaf level. At the whole-plant level, light use efficiency showed a strong positive linear relationship with *P*_gmax_. Notable was the variation in total Chl, Chl *b*, and Chl *a*/*b* ratio at the measurement dates, which tended to combine the characteristics of both high and low light-grown plants. Overall, *A*. *cruentus* and *A*. *hybridus* were superior to *A*. *dubius* and *A*. *hypochondriacus* with respect to the *P*_gmax_, leaf traits, and light use efficiency. Thus, *A*. *hybridus* and *A*. *cruentus* tend to be more efficient in carbon acquisition. These findings highlight the correlation between leaf photosynthetic characteristics, other leaf traits, and whole plant productivity in amaranths. Future studies may explore more species and accessions of *Amaranthus* spp.at different locations or light environments.

## Supporting information

S1 Fig Images of the *Amaranthus* species *(A*. *hybridus*, *A*. *dubius*, *A*. *hypochondriacus and A*. *cruentus)* studied. (DOCX)

S2 Fig Net Photosynthetic light response curves (A-D) and the corresponding stomatal conductance response (*g*_s_; E, F) at each light (Photosynthetic Photon Flux Density; PPFD) level in youngest fully expanded leaves of *A*. *hybridus*, *A*. *dubius*, *A*. *hypochondriacus*, and *A*. *cruentus* at three measurement dates (M). Measurement dates: M1 = May 7, 2014 (50 DAS); M2 = May 12, 2014 (55 DAS); and M3 = May 20, 2014 (63 DAS). DAS denotes days after sowing. Measurements were taken with the Licor-6400. Each curve for the measurement dates is an average of two biological replications (*n* = 2). Bars represent ± SD.
(TIF)

S1 Table Data collection dates and the corresponding days after sowing. (DOCX)

S2 Table Analysis of variance table and the effect size measure, omega squared (ω^2^) for the parameters of the net photosynthetic light response curves. *P*_gmax_ ‒ maximum gross photosynthetic rate (μmol (CO_2_) m^–2^ s^–1^); apparent quantum yield at zero PPFD (α(*I*_0_), μmol (CO_2_) μmol (photon)^–1^); dark respiration rate (*R*_D_, μmol (CO_2_) m^–2^ s^–1^); convexity (θ); Df: degrees of freedom; SS: sum of squares; MS = Mean squares.
(DOCX)

S3 Table Analysis of variance table and the effect size measure, omega squared (ω^2^) for leaf traits. Stomatal conductance (*g*_s_); total chlorophyll (total Chl); chlorophyll *a* (Chl *a*); chlorophyll *b* (Chl *b*); chlorophyll a to chlorophyll b ratio (Chl *a*/*b*); carotenoids (Car); nitrogen content per unit area (N_a_); leaf dry mass (LDM); leaf area (LA).
(DOCX)

S4 Table Mean comparison test (Tukey Honest Significant Difference) of measurement dates effect on chlorophyll *b* and total chlorophyll content. *n* = 48.
(DOCX)

S5 Table Mean comparison test (Tukey Honest Significant Difference) of the interaction effect of species and measurement dates on chlorophyll *a* and chlorophyll *b ratio*. *n* = 48.
(DOCX)

S6 Table Pearson’s correlation coefficients (*r*) and statistical significance for maximum gross photosynthetic rate and leaf traits in four amaranth species (*A*. *hybridus*, *A*. *dubius*, *A*. *hypochondriacus* and *A*. *cruentus*). *P*_gmax_, maximum gross photosynthetic rate (μmol (CO_2_) m^–2^ s^–1^); *g*_s_, Stomatal conductance (mmol m^-2^s^-1^); N_a_, nitrogen content per unit area (g m^-2^); Car-carotenoids (mmol m^-2^s^-1^); total Chl, Total chlorophyll (mmol m^-2^s^-1^); Chl *a*, chlorophyll *a* (mmol m^-2^s^-1^); Chl *b*, Chlorophyll *b* (mmol m^-2^s^-1^). Values represent Pearson’s correlation coefficient (*r*). Significance at *P*: <0.001***; <0.01**; <0.05*; NS—not significant.
(DOCX)
