# Supplementary material for: Interspecific variation in leaf traits, photosynthetic light response, and whole-plant productivity in amaranths (Amaranthus spp. L.)
Source: PLoS One. 2022 Jun 30;17(6):e0270674. doi: 10.1371/journal.pone.0270674 (PMC9246199; doi:10.1371/journal.pone.0270674)
Supplement: S1 Fig — (DOCX) [file pone.0270674.s001.docx]

| 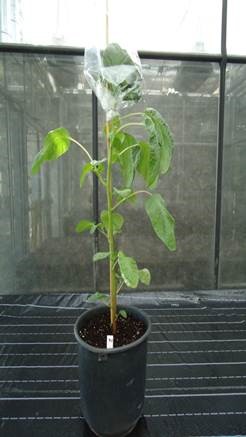   1. *A. hybridus* (IP7) | 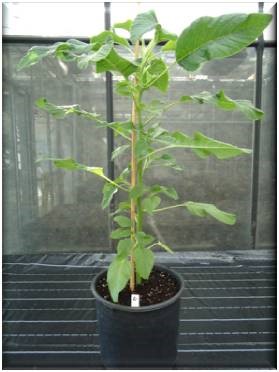   1. *A.dubius* (Mombo 2) |
| --- | --- |
| 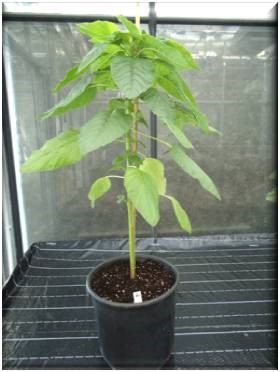   1. *A.hypochondriacus* (TZ-SMN-102) | 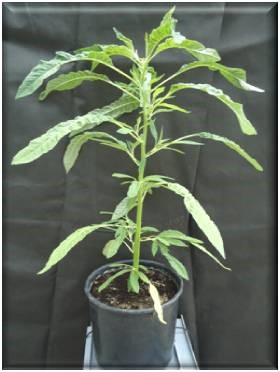   1. *A.cruentus* (Ex-Zim/Madiira 1) |

**S1 Fig. Images of the four *Amaranthus species (A. hybridus, A. dubius, A. hypochondriacus and A. cruentus)* studied.**
