# Supplementary figures and images for: Interspecific variation in leaf traits, photosynthetic light response, and whole-plant productivity in amaranths (Amaranthus spp. L.)
Source: PLoS One. 2022 Jun 30;17(6):e0270674. doi: 10.1371/journal.pone.0270674 (PMC9246199; doi:10.1371/journal.pone.0270674)

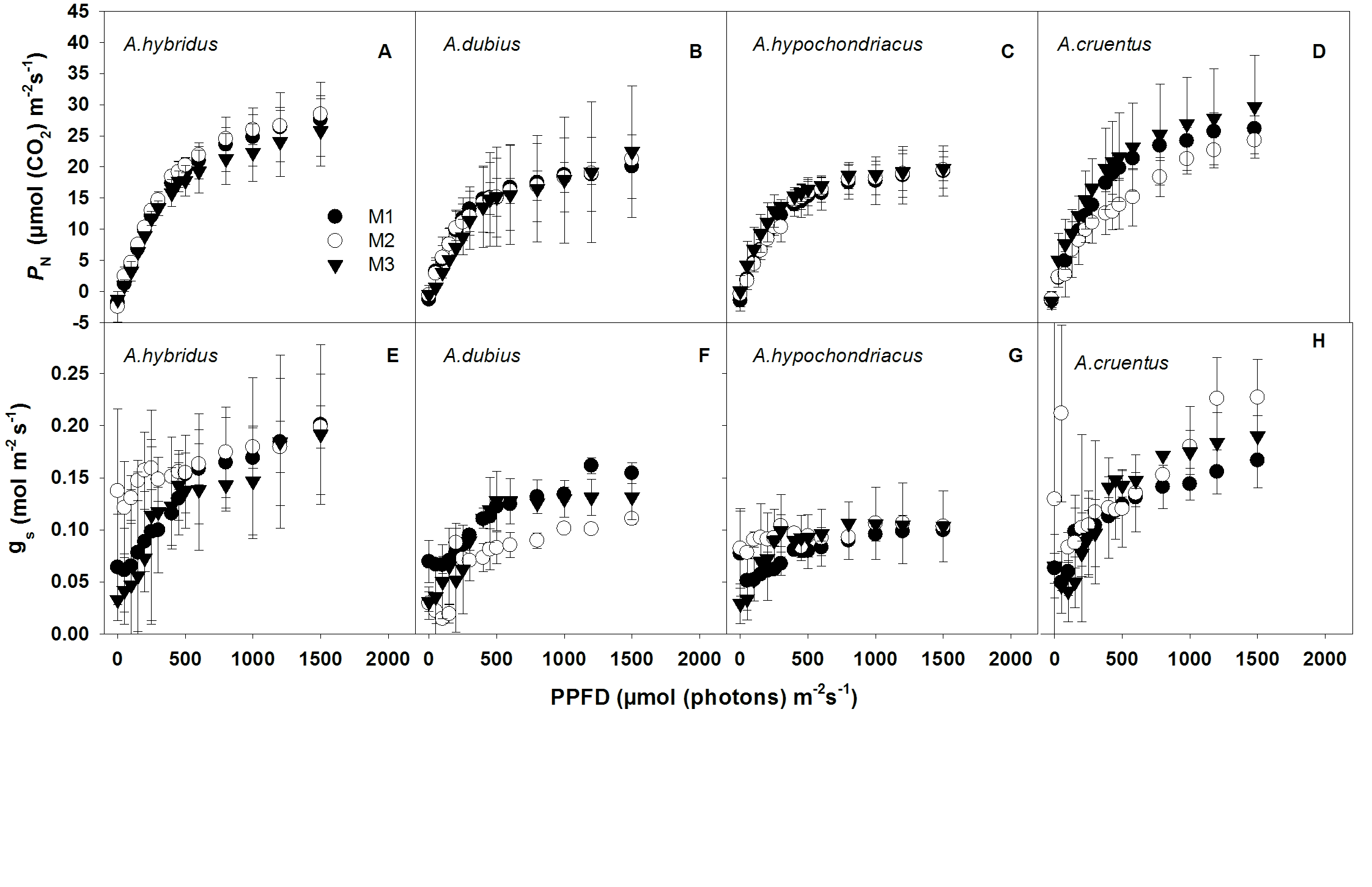

Supplement: S2 Fig — Net Photosynthetic light response curves (A-D) and the corresponding stomatal conductance response (gs; E, F) at each light (Photosynthetic Photon Flux Density; PPFD) level in youngest fully expanded leaves of A. hybridus, A. dubius, A. hypochondriacus, and A. cruentus at three measurement dates (M). Measurement dates: M1 = May 7, 2014 (50 DAS); M2 = May 12, 2014 (55 DAS); and M3 = May 20, 2014 (63 DAS). DAS denotes days after sowing. Measurements were taken with the Licor-6400. Each curve for the measurement dates is an average of two biological replications (n = 2). Bars represent ± SD. (TIF) [file pone.0270674.s002.tif]
