# Supplementary material for: Interspecific variation in leaf traits, photosynthetic light response, and whole-plant productivity in amaranths (Amaranthus spp. L.)
Source: PLoS One. 2022 Jun 30;17(6):e0270674. doi: 10.1371/journal.pone.0270674 (PMC9246199; doi:10.1371/journal.pone.0270674)
Supplement: S1 Table — (DOCX) [file pone.0270674.s003.docx]

**S1 Table. Data collection dates and the corresponding days after sowing.**

| Measurement Dates (2014) | Days after sowing (DAS) |
| --- | --- |
| 15 April | 28 |
| 29 April | 42 |
| 07 May | 50 |
| 12 May | 55 |
| 20 May | 63 |
