# Supplementary material for: Interspecific variation in leaf traits, photosynthetic light response, and whole-plant productivity in amaranths (Amaranthus spp. L.)
Source: PLoS One. 2022 Jun 30;17(6):e0270674. doi: 10.1371/journal.pone.0270674 (PMC9246199; doi:10.1371/journal.pone.0270674)
Supplement: S2 Table — Pgmax ‒ maximum gross photosynthetic rate (μmol (CO2) m–2 s–1); apparent quantum yield at zero PPFD (α(I0), μmol (CO2) μmol (photon)–1); dark respiration rate (RD, μmol (CO2) m–2 s–1); convexity (θ); Df: degrees of freedom; SS: sum of squares; MS = Mean squares. (DOCX) [file pone.0270674.s004.docx]

**S2 Table. Analysis of variance table with the effect size measure, omega squared (ω^2^) for the parameters of the net photosynthetic light response curves**.

| Parameters | Source | Test of effects | | | | | Measure of effect size |
| --- | --- | --- | --- | --- | --- | --- | --- |
|  |  | *Df* | *SS* | *MS* | *F*-values | *P*-values | ω^2^ |
| *P*_gmax_ | Species | 3 | 776.921 | 258.974 | 12.097 | <0.001 | 0.484 |
|  | Measurement dates (Md) | 2 | 7.813 | 3.906 | 0.182 | 0.836 | 0.000 |
|  | Species x Md | 6 | 109.935 | 18.323 | 0.856 | 0.555 | 0.000 |
|  | Replications | 1 | 319.471 | 319.471 | 14.923 | 0.003 | 0.039 |
| α(*I*_0_) | Species | 3 | 0.000 | 0.000 | 0.139 | 0.934 | 0.000 |
|  | Md | 2 | 0.001 | 0.001 | 0.937 | 0.421 | 0.000 |
|  | Species x Md | 6 | 0.005 | 0.001 | 1.144 | 0.399 | 0.037 |
|  | Replications | 1 | 0.001 | 0.001 | 2.050 | 0.180 | 0.045 |
| *R*_D_ | Species | 3 | 4.307 | 1.436 | 1.160 | 0.369 | 0.029 |
|  | Md | 2 | 0.232 | 0.116 | 0.094 | 0.911 | 0.000 |
|  | Species x Md | 6 | 1.169 | 0.195 | 0.157 | 0.983 | 0.000 |
|  | Replications | 1 | 0.063 | 0.063 | 0.051 | 0.826 | 0.000 |
| θ | Species | 3 | 0.346 | 0.115 | 0.737 | 0.551 | 0.000 |
|  | Md | 2 | 0.282 | 0.141 | 0.902 | 0.434 | 0.000 |
|  | Species x Md | 6 | 0.521 | 0.087 | 0.555 | 0.757 | 0.000 |
|  | Replications | 1 | 0.000 | 0.000 | 0.001 | 0.971 | 0.000 |

*P*_gmax_ ‒ maximum gross photosynthetic rate (μmol (CO_2_) m^–2^ s^–1^); apparent quantum yield at zero PPFD (α(*I*_0_), μmol (CO_2_) μmol (photon)^–1^); dark respiration rate (*R*_D,_ μmol (CO_2_) m^–2^ s^–1^), convexity (θ); Df: degrees of freedom; SS: sums of squares; MS = Mean squares. n = 24
