# Supplementary material for: Interspecific variation in leaf traits, photosynthetic light response, and whole-plant productivity in amaranths (Amaranthus spp. L.)
Source: PLoS One. 2022 Jun 30;17(6):e0270674. doi: 10.1371/journal.pone.0270674 (PMC9246199; doi:10.1371/journal.pone.0270674)
Supplement: S3 Table — Stomatal conductance (gs); total chlorophyll (total Chl); chlorophyll a (Chl a); chlorophyll b (Chl b); chlorophyll a to chlorophyll b ratio (Chl a/b); carotenoids (Car); nitrogen content per unit area (Na); leaf dry mass (LDM); leaf area (LA). (DOCX) [file pone.0270674.s005.docx]

**S3 Table. Analysis of variance table and the effect size measure, omega squared (ω^2^) for leaf traits**.

| Leaf traits | Source of variation | *Df* | *SS* | *MS* | *F*-values | *P*-values | ω^2^ |
| --- | --- | --- | --- | --- | --- | --- | --- |
| *g*_s_ (mol m^-2^ s^-1^), mean from all PPFD levels | Species (Spp) | 3 | 0.010 | 0.003 | 4.983 | 0.020* | 0.341 |
|  | Measurement dates (Md) | 2 | 0.001 | 0.001 | 0.858 | 0.451 | 0.000 |
|  | Spp x Md | 6 | 0.004 | 0.001 | 1.026 | 0.458 | 0.004 |
|  | Reps | 1 | 0.000 | 0.000 | 0.215 | 0.652 | 0.000 |
| *g*_s_ (mol m^-2^ s^-1^) @ PPFD 1500 μmol m^–2^ s^–1^ | Spp | 3 | 0.039 | 0.013 | 8.266 | 0.004* | 0.490 |
|  | Md | 2 | 8.03E-05 | 4.01E-05 | 0.026 | 0.974 | 0.000 |
|  | Spp x Md | 6 | 0.006 | 0.001 | 0.649 | 0.691 | 0.000 |
|  | Reps | 1 | 0.006 | 0.000 | 3.721 | 0.079 | 0.061 |
| Total Chl (mmol m^-2^) | Spp | 3 | 0.366 | 0.122 | 9.506 | <0.001* | 0.322 |
|  | Md | 2 | 0.102 | 0.051 | 3.968 | 0.029* | 0.075 |
|  | Spp x Md | 6 | 0.027 | 0.005 | 0.351 | 0.904 | 0.000 |
|  | Reps | 3 | 0.086 | 0.029 | 2.233 | 0.103 | 0.047 |
| Chl *a* (mmol m^-2^) | Spp | 3 | 0.205 | 0.068 | 8.200 | 0.000* | 0.295 |
|  | Md | 2 | 0.046 | 0.023 | 2.786 | 0.076 | 0.049 |
|  | Spp x Md | 6 | 0.019 | 0.003 | 0.381 | 0.886 | 0.000 |
|  | Reps | 3 | 0.056 | 0.019 | 2.229 | 0.103 | 0.050 |
| Chl *b* (mmol m^-2^) | Spp | 3 | 0.023 | 0.008 | 14.978 | <0.001* | 0.379 |
|  | Md | 2 | 0.012 | 0.006 | 11.294 | <0.001* | 0.187 |
|  | Spp x Md | 6 | 0.001 | 0.000 | 0.421 | 0.860 | 0.000 |
|  | Reps | 3 | 0.003 | 0.001 | 2.105 | 0.118 | 0.003 |
| Chl *a*/*b* | Spp | 3 | 5.449 | 1.816 | 7.701 | <0.001* | 0.162 |
|  | Md | 2 | 9.892 | 4.946 | 20.973 | <0.001* | 0.323 |
|  | Spp x Md | 6 | 5.528 | 0.921 | 3.907 | 0.005* | 0.141 |
|  | Reps | 3 | 0.336 | 0.112 | 0.475 | 0.701 | 0.000 |
| Car (mmol m^-2^) | Spp | 3 | 0.030 | 0.010 | 8.202 | <0.001* | 0.298 |
|  | Md | 2 | 0.004 | 0.002 | 1.479 | 0.243 | 0.013 |
|  | Spp x Md | 6 | 0.005 | 0.001 | 0.642 | 0.696 | 0.000 |
|  | Reps | 3 | 0.009 | 0.003 | 2.399 | 0.086 | 0.058 |
| N_a_ (g m^-2^) | Spp | 3 | 0.930 | 0.310 | 3.764 | 0.020* | 0.147 |
|  | Md | 2 | 0.149 | 0.074 | 0.904 | 0.415 | 0.000 |
|  | Spp x Md | 6 | 0.118 | 0.020 | 0.239 | 0.961 | 0.000 |
|  | Reps | 3 | 0.635 | 0.212 | 2.568 | 0.071 | 0.084 |
| LDM (g) | Spp | 3 | 1.002 | 0.334 | 9.826 | <0.001* | 0.335 |
|  | Md | 2 | 0.077 | 0.038 | 1.127 | 0.336 | 0.003 |
|  | Spp x Md | 6 | 0.371 | 0.062 | 1.818 | 0.126 | 0.062 |
|  | Reps | 3 | 0.085 | 0.028 | 0.830 | 0.487 | 0.000 |
| LA (cm^2^) | Spp | 3 | 28400.504 | 9466.835 | 20.069 | <0.001* | 0.510 |
|  | Md | 2 | 1271.700 | 635.850 | 1.348 | 0.274 | 0.006 |
|  | Spp x Md | 6 | 6196.964 | 1032.827 | 2.190 | 0.069 | 0.064 |
|  | Reps | 3 | 964.392 | 321.464 | 0.681 | 0.570 | 0.000 |

Stomatal conductance (*g*_s_); total chlorophyll (total Chl); chlorophyll *a* (Chl *a*); chlorophyll *b* (Chl *b*); chlorophyll a to chlorophyll b ratio (Chl *a*/*b*); carotenoids (Car); nitrogen content per unit area (N_a_); leaf dry mass (LDM); leaf area (LA). g_s_ (n = 24); other leaf traits (n = 48)
