# Supplementary material for: Interspecific variation in leaf traits, photosynthetic light response, and whole-plant productivity in amaranths (Amaranthus spp. L.)
Source: PLoS One. 2022 Jun 30;17(6):e0270674. doi: 10.1371/journal.pone.0270674 (PMC9246199; doi:10.1371/journal.pone.0270674)
Supplement: S4 Table — n = 48. (DOCX) [file pone.0270674.s006.docx]

**S4 Table**. **Mean comparison test (Tukey Honest Significant Difference) of measurement dates effect on chlorophyll *b* and total chlorophyll content.**

| Measurements | Measurement dates (2014) | Chl *b* (mmol m^–2^ ) | Total Chl (mmol m^–2^ ) |
| --- | --- | --- | --- |
| 1 | 07 May (50 DAS) | 0.13^A^ (0.12-0.14) | 0.61^A^ (0.55-0.67) |
| 2 | 12 May (55 DAS) | 0.12^A^ (0.10-0.13) | 0.60^AB^ (0.54-0.66) |
| 3 | 20 May (63 DAS) | 0.09^B^ (0.08-0.10) | 0.51^B^ (0.45-0.56) |
| SEM | - | 0.01 | 0.03 |

Levels not connected by the same letters are significantly different (p = 0.05). Bracketed values are the confidence intervals (95%). Chlorophyll *b* (Chl *b*)*,* total chlorophyll content (total Chl). DAS denotes days after sowing. n = 48
