# Supplementary material for: Interspecific variation in leaf traits, photosynthetic light response, and whole-plant productivity in amaranths (Amaranthus spp. L.)
Source: PLoS One. 2022 Jun 30;17(6):e0270674. doi: 10.1371/journal.pone.0270674 (PMC9246199; doi:10.1371/journal.pone.0270674)
Supplement: S5 Table — n = 48. (DOCX) [file pone.0270674.s007.docx]

**S5 Table**. **Mean comparison test (Tukey Honest Significant Difference) of the interaction effect of species and measurement dates on chlorophyll *a* and chlorophyll *b ratio.***

| Species | Chlorophyll a and chlorophyll b ratio (95% CI)  (n = 48) | | |
| --- | --- | --- | --- |
|  | Measurement dates (2014) | | |
|  | 7 May (50 DAS) | 12 May (55 DAS) | 20 May (63 DAS) |
| *A.hybridus* | 3.61^C^ (3.11-4.10) | 3.98^BC^ (3.49-4.47) | 3.83^BC^ (3.33-4.32) |
| *A.dubius* | 3.77^BC^ (3.28-4.27) | 4.43^BC^ (3.94-4.93) | 6.03^A^ (5.54-6.53) |
| *A.hypochondriacus* | 3.94^BC^ (3.44-4,43) | 4.45^BC^ (3.96-4.95) | 4.64^BC^ (4.14-5.13) |
| *A.cruentus* | 3.63^C^ (3.14-4.12) | 4.06^BC^ (3.56-4.55) | 4.89^AB^ (4.40-5.39) |
| SEM | 0.243 | | |

Levels not connected by the same letters are significantly different (p = 0.05). DAS, days after sowing.
