# Supplementary material for: Interspecific variation in leaf traits, photosynthetic light response, and whole-plant productivity in amaranths (Amaranthus spp. L.)
Source: PLoS One. 2022 Jun 30;17(6):e0270674. doi: 10.1371/journal.pone.0270674 (PMC9246199; doi:10.1371/journal.pone.0270674)
Supplement: S6 Table — Pgmax, maximum gross photosynthetic rate (μmol (CO2) m–2 s–1); gs, Stomatal conductance (mmol m-2s-1); Na, nitrogen content per unit area (g m-2); Car-carotenoids (mmol m-2s-1); total Chl, Total chlorophyll (mmol m-2s-1); Chl a, chlorophyll a (mmol m-2s-1); Chl b, Chlorophyll b (mmol m-2s-1). Values represent Pearson’s correlation coefficient (r). Significance at P: <0.001***; <0.01**; <0.05*; NS—not significant. (DOCX) [file pone.0270674.s008.docx]

**S6 Table**. **Pearson’s correlation coefficients (*r*) and the statistical significance for maximum gross photosynthesis rate and leaf traits in four amaranth species (*A. hybridus*, *A. dubius*, *A. hypochondriacus* and *A. cruentus*).**

| **Parsons’ correlation coefficient ( *r*)** | | | | | | | | |
| --- | --- | --- | --- | --- | --- | --- | --- | --- |
|  | *P*_gmax_ | g_s_ | Na | Car | Total Chl | Chl *a* | Chl *b* | LA |
| *P*_gmax_ | 1 |  |  |  |  |  |  |  |
| g_s_ | 0.873*** | 1 |  |  |  |  |  |  |
| N_a_ | 0.706** | 0.602* | 1 |  |  |  |  |  |
| Car | 0.614* | 0.699* | 0.375 ^NS^ | 1 |  |  |  |  |
| Total Chl | 0.594* | 0.672* | 0.469 ^NS^ | 0.955*** | 1 |  |  |  |
| Chl *a* | 0.620* | 0.690* | 0.454 ^NS^ | 0.969*** | 0.996*** | 1 |  |  |
| Chl *b* | 0.498 ^NS^ | 0.594* | 0.490 ^NS^ | 0.880*** | 0.971*** | 0.947*** | 1 |  |
| LA | -0.655* | -0.658* | -0.544 ^NS^ | -0.632* | -0.610* | -0.607* | -0.595* | 1 |

*P*_gmax_, maximum gross photosynthetic rate (μmol (CO_2_) m^–2^ s^–1^); *g*_s_, Stomatal conductance (mmol m^-2^s^-1^); N_a_, nitrogen content per unit area (g m^-2^); Car-carotenoids (mmol m^-2^s^-1^); total Chl, Total chlorophyll (mmol m^-2^s^-1^); Chl *a*, chlorophyll *a* (mmol m^-2^s^-1^); Chl *b*, Chlorophyll *b* (mmol m^-2^s^-1^). Values represent Pearson’s correlation coefficient (*r*). Significance at *P*: <0.001***; <0.01**; <0.05*; NS - not significant.
